# Seasonable Thoughts

**Published:** 1909-12-25

**Authors:** 


### The Hospital

A Journal of

The Medical Sciences and Hospital Administration.

New Series. No. 149, Vol. VI. [No. 1221, Vol. XLVII.] SATURDAY, DECEMBER 25, 1909.

### SEASONABLE THOUGHTS.

Some relaxation from ordinary professional cares is permissible at this time of year. The general practitioner, however hard worked, will find a spare moment in which to put away from him, wholly and heartily, all thoughts of his work, and devote himself to the seasonable thoughts that immemorial custom has decreed should occupy the meditative at the close of the year. We may assert, with Charles Lamb, that no man who is introspective, even painfully so, can enjoy festival days without a pang. It is a dictum the truth of which will be easily apparent to every member of the profession, and to every thoughtful man or woman who interests him or herself in institutional or charitable work. At such times when introspective or retrospective meditation rules, the cup of whatever is pleasurable is made bitter by the thought of something that in the past days of the year was left undone or done slovenly; sins of commission and of omission, the tabulation on the debit side of the balance-sheet blighting, and almost effacing, the opposite figures. The total valuation of the misery or anguish of mind resulting from such mental auditing will depend largely upon the psychological factors influencing the individual at the time when this annual stocktaking is in progress. Few of us will venture to assert that under the cheerful and cheering influences of Christmastide, the damage to individual self-respect will be incalculably less than if the self-analysing process had been done on midsummer day or the commencement, say, of the Michaelmas term. At the same time, no matter where or when it is done, such self-analysis, provided it be thorough, impartial, and just, must, in the case of medical men at least, be conducive to some sadness of mind. As members of a profession that should be in the front rank of reformers, in the van of social and hygienic progress, medical men have much to answer for. A great responsibility rests upon them as exemplars. Hitherto most of us have been under the impression that our moral and intellectual duties are abundantly accomplished when we have confined ourselves to the easy rôles of preceptors. Every doctor has an ingrained propensity for lecturing: it is a fault that was matured in him during his student days and that is kept vigorously alive by the frequent opportunities he has, in practice, of laying down the law to cowering laymen. Few of the profession, on the other hand, have the courage to practise what they preach. We may not be altogether as immoral as Mr. Bernard Shaw maintains that generically all doctors are; we may not condemn quackery in public and live on Somebody's Patent Pills in private, for all that Mr. Maarten Maartens may assert to the contrary, and we may scruple to preach what we do not believe, as effectually as Gil Blas of Santillane scrupled to do what he deemed dishonest. But, in little things especially, we are hopelessly untrue to our ideals, and it is as well that we should searchingly investigate our position and arrive at a definite conclusion as to the extent of our deficiencies as exemplars.

We depart in this number of The Hospital from our usual Christmas custom, and, under the impression that the average practitioner cares nothing for clinics at this festive time, we have attempted to make our Christmas issue a number that may to some extent help the reader to realise what should be put down to the credit, what to the debit side of such an account as we have hinted at above. During the past year there have been many grave and important problems which have engaged the attention of all who are interested in sociological questions. The majority of these vital questions concern us as medical men, and as intellectual leaders of the public (we need neither blush nor apology for arrogating to the medical profession so high a position as this). They concern us closely not only because they are matters upon which scientifically trained intelligence may reasonably express opinions of greater value than those emanating from laymen whose judgments may be vitiated and impaired by sentimental, political, or religious feelings, but more especially because many of them are national questions. In this issue we have tried to epitomise the leading problems, but it would take far more space than we can allow to do justice to the majority of these interesting questions of the day. That the brief summaries here published may stimulate general practitioners to further inquiries in these problems is all that we can hope for, and with that hope expressed we send out this number, confident that the average medical man needs only to be shown the importance of his position as a public exemplar to realise his duties to the public, and do them as faithfully and as conscientiously as he now performs those he owes to his individual patients. At this season it is surely not too much to ask him for a few moments to consider the relation between our hospitals and the public, the government and the profession, and the profession and the laity, together with the numerous side issues involved in such a consideration.

We venture to suggest that such thoughts as we propose may legitimately be classed under the heading which caps this article. They are truly seasonable, and their consideration will be doubly instructive and beneficial at this time, when "questions of the day" are likely to be discussed more than at any other. For these reasons we commend to our readers the special articles which appear in this number, and in wishing them all the compliments of the season we trust sincerely that their Christmas-tide may not be made less bright and less happy by the reflection that there has been very little real progress in the past year, and that some of the hardest fights are yet to come, some of the most difficult situations yet to be faced, if the medical profession is to do its duty to the public in the future.

